# A Preliminary Study of the Effect of 3D Printing Orientation on Mechanical Properties and Fracture of Samples Made from AlSi10Mg

**DOI:** 10.3390/ma18235294

**Published:** 2025-11-24

**Authors:** Katarina Monkova, Marianthi Bouzouni, George A. Pantazopoulos, Anagnostis I. Toulfatzis, Sofia Papadopoulou

**Affiliations:** 1 Faculty of Manufacturing Technologies with a Seat in Presov, Technical University in Kosice, Sturova 31, 080 01 Presov, Slovakia; 2 Faculty of Technology, Tomas Bata University in Zlin, Vavreckova 5669, 760 01 Zlin, Czech Republic; 3 ELKEME Hellenic Research Centre for Metals S.A., 61st km Athens—Lamia National Road, 32011 Oinofyta, Greece; gpantaz@elkeme.vionet.gr (G.A.P.); atoulfatzis@elkeme.vionet.gr (A.I.T.); spapadopoulou@elkeme.gr (S.P.)

**Keywords:** AlSi10Mg alloy, mechanical properties, tensile testing, SLM, 3D printing specimens’ orientation, fractography, microscopy analysis, thermodynamic simulation

## Abstract

The significant advancement in additive technologies has made it possible to manufacture metal components in diverse shapes and sizes. Despite this progress, numerous processes and phenomena, along with the implications of producing components layer by layer on their performance under stress, remain inadequately explored. These factors not only affect microstructure but subsequently also the mechanical properties. The positioning of objects within the 3D printer’s workspace can thus significantly play a crucial role in their operational functionality, reliability, and safety of the equipment in an application. This article studies anisotropic properties and the influence of the printing orientation of aluminum alloy (AlSi10Mg) cylindrical tensile samples fabricated through an additive approach on their mechanical properties under tensile loading. Tensile testing of specimens covering seven different spatial orientations in the workspace of a 3D printing machine was performed according to ISO 6892-1 international standard. Minimum and maximum tensile properties (yield and ultimate tensile strength) have been observed in Y-sample and X-sample series, respectively. In contrast, elastic modulus of the 3D printed specimens was minimal for X-sample series, and maximal for Y-sample series. Fracture surfaces of the samples in seven basic spatial orientations were evaluated in synergy with the mechanical testing results determined by optical, electron microscopy, and electron backscatter diffraction (EBSD) textural analysis to find correlation between the strength of the samples and the orientation of grains, their size and morphology. Furthermore, thermodynamic and Scheil–Gulliver simulation has been employed in order to explain the formation of intermetallic phases during additive manufacturing and further justifying observations in microstructure and mechanical properties. The disparity in texture intensity between these regions for samples Y(3) is likely responsible for localized mechanical incompatibilities and strain heterogeneity, resulting in preferential crack paths and reduced mechanical strength compared to the sample Z(3), which presented a more randomized orientation distribution with less distinguishable texture zones, enabling better strain accommodation and more uniform plastic deformation, which correlates with its higher tensile and yield strength.

## 1. Introduction

Processes of Metal Additive Manufacturing (AM), exemplified by Selective Laser Melting (SLM), have revolutionized the production of intricate 3D structures, offering extensive design flexibility. Ongoing research in this domain holds significant promise, with transformative implications for diverse scientific and industrial applications. Various sectors are now leveraging AM technologies to create complex structures, achieving objectives like lightweighting, enhanced functionality, and part count reduction. The potential of AM extends to cost reduction and accelerated design-to-manufacture timelines.

A noteworthy advantage is the ability to consolidate components within assemblies, reducing dependence on mechanical fixtures and supplementary production operations. This opens avenues for innovative and efficient product design, demonstrating the transformative potential of Metal AM in pushing the boundaries of traditional manufacturing capabilities [1,2].

Depending on the chosen AM approach, designers can capitalize on distinct advantages. Evolutionary strategy modifications of existing products or innovative approaches that introduce unprecedented functionalities are viable options. The core rationale for implementing AM lies in design freedom, enabling a significant enhancement in the effectiveness and functionality of existing designs. AM facilitates the incorporation of intricate features typically constrained by conventional manufacturing techniques. This includes implementing complex core structures or channels, such as lattices, thereby supporting lightweighting through topology optimization. In addition, AM enables the production of multifunctional or multi-material devices. In terms of materials, it could solve the problem of processing aluminum alloys by conventional machining, which can be very important for complex-shaped parts used mainly in the aerospace, aviation and automotive industries [3,4,5].

However, despite the advantages of additively manufactured samples, there are still many challenges and unanswered questions that need to be explored and answered to ensure that safety and reliability remain a priority in the operation of aircraft and automobiles, as well as other devices.

## 2. State of the Art

The significant advancement in additive technologies has made it possible to manufacture metal components in diverse shapes and sizes. Despite this progress, numerous processes and phenomena, along with the implications of producing components layer by layer on their performance under stress, remain inadequately explored. These factors not only affect microstructure but subsequently also the mechanical properties. The positioning of objects within the 3D printer’s workspace can thus significantly play a crucial role in their operational functionality, reliability, and safety of the equipment in an application. Scientists and experts have already addressed the influence of a sample’s orientation on its mechanical and structural properties, looking at this problem from different perspectives. Bean et al. [6] studied the anisotropy of Inconel 718 produced by SLM in three directions. They found that Z samples showed the lowest strength and the highest elongation. On the contrary, XY samples displayed the highest strength and the lowest elongation. Optical microscopy, SEM, and EBSD analyses were also performed to investigate microstructural alteration that could account for the differences in mechanical characteristics. EBSD analysis of anisotropy indicated a preferred grain texture and twinning orientation consistent with the growth direction, which may have contributed to the anisotropic ductility enhancement.

3D printing orientation on tensile behaviour and fracture mechanisms of additively produced samples from Inconel 718 were analysed also by Monkova at al. [7] Specimens produced in seven directions were fabricated by DMLS technology and their tensile characteristics were assessed. In this research the highest tensile strength and elongation at fracture were observed at XZ orientation, and that the directionality of the microstructure affects the fracture mechanism through alteration of the crack propagation.

Monkova et al. [8] also studied MS1 steel bone samples produced by AM in five directions such as X, Y, XY, XYZ and Z, while three states of samples were considered: (1) as-printed state without additional heat treatment; (2) samples after annealing and (3) samples after hardening. Moreover, the authors compared tensile properties of samples produced in two different institutions and tested by two independent laboratories with properties of conventionally produced 1.2709 steel. The research proved that samples produced in X, Y, Z directions displayed relatively isotropic strength values, while the fracture surfaces and the fracture mechanism were primarily related to heat treatment and not to the 3D-print direction. The valuable findings have been related to the dominance of both strength characteristics (yield and ultimate strength) in the YZ direction for the samples in as-printed state.

In the research of Zhang [9] a Ni-based alloy GH4099 manufactured by SLM followed by heat treatment in which the microstructure and mechanical properties were investigated. Samples manufactured perpendicular and parallel to the layering direction showed minimal differences in tensile strength, but notable anisotropy was revealed by large differences in elongation. At room temperature, the alloy’s deformation mechanism was consistent, with cracks forming within grains and grain boundaries acting as barriers to dislocations. At high temperatures, fractures occur at grain boundaries due to carbide growth. Loading along different grain axes produced distinct deformation mechanisms.

Singh et al. [10] studied additively fabricated flat samples of Ti-6Al-4V alloy, while some of them were heat treated to relieve residual stresses, which were reduced but at the expense of strength loss. Samples perpendicular to the build direction (0°) showed high tensile strength (1202 MPa) and microhardness but low ductility. Vertical samples (90°) had the greatest elongation (8%) but suffered from low density and high residual stress (163 MPa). Inclined samples (45°) showed the lowest residual tensile stress after heating. Defects like pores, unmelted powder, and cracks reduce tensile strength and increased surface roughness.

Similarly, Barba [11] with his team investigated Ti-6Al-4V specimen size and orientation effect on their mechanical properties, while strength and ductility were evaluated. They found out that the weakest tensile strength showed the samples printed in the ZZ direction.

Among the materials garnering substantial attention in SLM research, aluminium alloys stand out, particularly valued in high-value applications. They find extensive use across diverse industries due to their favourable combination of strength and density, coupled with cost-effectiveness. They have become a preferred choice in applications at which achieving both high performance and lightweight characteristics is crucial. Aluminium alloy parts have gained traction in consumer, aerospace, and automotive products [12]. SLM’s applicability extends to challenging-to-machine or form aluminium alloys, addressing issues encountered with alloys like, e.g., AA-6xxx, known for their high content of hard intermetallic particles that pose difficulties during traditional forming processes [13].

One of the distinct advantages of SLM in processing aluminium, especially cast alloys, lies in microstructural enhancement. Traditional strengthening methods for cast alloys involve refining their microstructures during casting through the addition of chemical modifiers. The rapid cooling rates inherent in SLM contribute to microstructural refinement during the manufacturing process [14], preserving the original chemical composition. This capability positions SLM as a suitable method for fabricating intricate structures with refined microstructures in castable aluminium alloys. On another hand, aluminium alloys pose notable challenges in their processing through Selective Laser Melting (SLM). The inherent properties of aluminium powders, such as their lightweight nature, high reflectivity, elevated thermal conductivity, and low laser absorptivity within the wavelength range of fibre lasers (1.06 µm [15]), commonly employed in SLM, contribute to these challenges. For a detailed exploration of these issues, readers are directed to [16].

The anisotropy of tensile properties of 3D printed AlSi10Mg alloy has been studied by very few researchers. One of them is, e.g., Kempen [17], who, however, studied flat samples of AlSi10Mg alloy made only in two directions, XY and Z, via SLM. A comparative analysis of the stress–strain curves for the two orientations revealed distinct hardening behaviors. Consequently, samples oriented in the XY direction exhibited lower elongation at break than those oriented in the Z direction. Additionally, tensile testing demonstrated a marked difference in ductility between parts fabricated along the XY axis and those produced along the Z axis.

**Figure 1:**
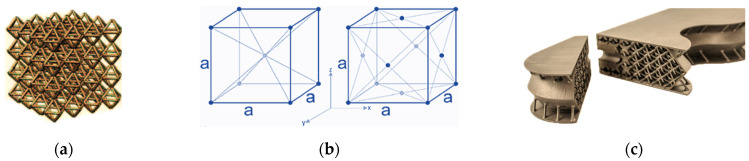
(**a**) An example of beams arranged differently in space creating porous lattice structures; (**b**) Principle of lattice structures of BCC (Body-Centered Cubic) and FCC (Face-Centered Cubic) types; (**c**) lightening the weight of a component by applying structures to its core.

The low weight of aluminium alloys makes them suitable for use in the aerospace and automotive industries. However, applications in these industries are looking for other ways to reduce weight, with porous materials appearing as one of the solutions, which have great prospects for their implementation in component cores. Due to these reasons, Selective Laser Melting (SLM), a prominent additive manufacturing technique, has proven itself not only in the production of fully volumetric bodies, but also in the production of metamaterial structures. In particular, the production of porous or cellular aluminium is notable for its lightweight properties and excellent deformability, making it an excellent candidate for applications such as, e.g., deformation/crash zones in automobiles. [18]. The walls and beams of such porous metamaterials are arranged differently in space and therefore must carry different loads. The struts or walls bounding the porous cells are oriented in different directions with respect to the direction of adding layers of material (building the body) as shown in Figure 1.

When producing bodies by laser powder bed fusion L-PBF (using DMLS, SLM or SLS technologies), the angle of inclination of the laser beam relative to the platform plane also changes, which, in combination with the orientation/position of the walls/barriers of such porous structures, can cause defects and consequently deviations in the resulting product properties due to different conditions of their construction (e.g., changing temperature gradient).

At the same time, however, the high demands of manufacturers on the safety and reliability of components manufactured by SLM technology from the AlSi10Mg alloy and the lack of knowledge about the behaviour of walls (struts, or parts of components) manufactured in different directions, motivated the authors to investigate the influence of 3D printing orientation on the tensile, textural and thermodynamic properties of AlSi10Mg samples. Knowledge of these properties can help designers prevent damage and unexpected failure of devices when they are put into operation.

The research provides insight into the mechanical and microstructural properties of the AlSi10Mg alloy produced in 7 basic directions of space by the SLM method including a view on fracture surfaces evaluation by optical, electron microscopy, and electron backscatter diffraction (EBSD) textural analysis to find correlation between strength of the samples and the orientation of grains, their size and morphology; as well as thermodynamic and Scheil–Gulliver simulation to explain formation of intermetallic phases during additive manufacturing and further justifying observations in microstructure and mechanical properties, has been lacking. It also points out the anisotropic properties of 3D printed materials, and identifies the causes of anisotropy, different behaviour of samples (and therefore failure) from different angles of view; it provides texture examination and points out the challenges with correct parameters and boundary conditions setup that need to be taken into account in 3D printed components before application into practice to avoid an unexpected/early failure. The findings will help designers in implementing the 3D printed material into real-world products and will contribute not only to the safety and reliability of their operation, but also to increasing their service life and preventing damage.

## 3. Materials and Methods

### 3.1. Samples Characteristics, Production and Testing

Identifying the causes of failure of 3D printed AlSi10Mg (EU: 3.2381, USA: A 360) alloy is an important aspect in preventing the failure rate of components manufactured by SLM technology and ensuring their reliable and safe operation. AlSi10Mg has increased strength and hardness compared to other aluminium alloys due to the presence of silicon and magnesium. AlSi10Mg fabricated with SLM shows fine microstructure and high strengths [19] in as-printed condition due to the very rapid melting and solidification.

**Figure 2:**
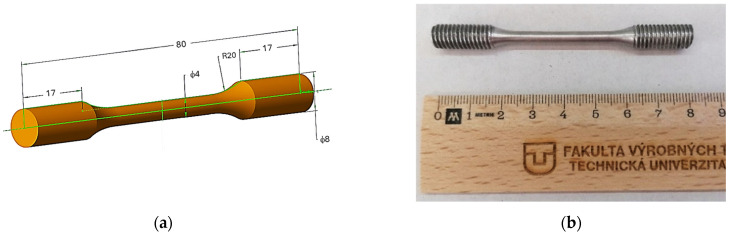
(**a**) Virtual 3D model showing the specimen dimensions; (**b**) a representative sample.

In the presented research, the behaviour of this material under load was studied via analysing the effect of 3D printed orientation of AlSi10Mg dog-bone type specimens on tensile, textural and thermodynamic properties. Declared properties given by the material datasheet of the alloy and the chemical composition [20] are listed in Table 1 and Table 2, while a typical sketch showing the specimen dimensions and a representative specimen is shown in Figure 2.

The rotational type of dog-bone type samples was selected to ensure that the shape (cross-section of the samples) would always be the same in each chosen direction. Since the different orientation of other types of samples in space, for example flat ones with a rectangular cross-section, and the associated different cross-sectional characteristics for different directions, are sensitive not only to the thermal processes occurring during production and subsequent heat treatment, but also, for example, to the assessment of stiffness; the chosen geometry was also to help mitigate differences between samples due to their placement in the working space of the 3D machine.

### 3.2. Production and Testing of Samples

**Figure 3:**
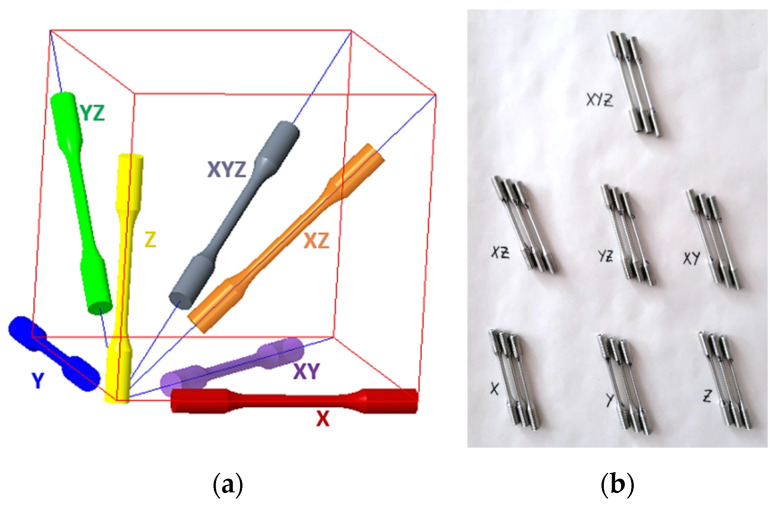
(**a**) Specification of seven orientations; (**b**) (3D printed AlSi10Mg specimens. (The lines shown in red delimit the basic orthogonal space, while the diagonals (both planar and spatial) are shown in blue.)

Three tensile specimens (3D printed AlSi10Mg) in each of seven (7) different orientations X, Y, Z, XY(A), XZ(B), YZ(C) and XYZ(U) of the 3D printer workspace were fabricated (Figure 3a), so that a total of twenty-one (21) dog bone specimens presented in Figure 3b were fabricated.

In the proposed coordinate system in Figure 3a, the horizontal surface formed by the XY axes represents the build platform and the Z axis represents the direction of layer deposition, the so-called build direction. A recoater blade is used to spread the metal powder across the build platform in the *Y*-axis direction, while scan direction in SLM refers to the orientation and path that the laser follows as it fuses each layer of powder.

The samples were made from powder material range of a grain diameter of 40–80 µm using SLM (Selective Laser Melting) technology and employing EP-M250 3D PRINTER (SHINING 3D Tech. Co., Ltd., Hangzhou, China) with fibre laser 500 W and Ar gas supply. Layer thickness of 30 µm was used according to the recommendation of the machine producer for the given material. Scan speed 2.5 m/s with scanning strategy Meander with 90° interlayer rotation and a spot size 70 µm were used. Hatch spacing (150 µm) was controlled by the proprietary Eplus 3D printing software (version EP-M250 3D printer) that allows using optimized scanning strategies to reduce print times. The samples were heat treated for stress relief at 300 °C in a convection air furnace with specimens on build plate, kept at the temperature for 2 h and then air cooled at a rate equal to air cooling in accordance with ASTM F3318-18 Condition SR1—Parts stress relieved. This specification covers additively manufactured AlSi10Mg (similar to DIN EN 1706/EN AC-43000) [21] parts using powder bed fusion such as laser melting. The specimens with support structures were cut off the platform using electrical discharge machining (EDM), sandblasted to remove the support structures and finished by grinding on a belt grinder.

Tensile tests were conducted on standard specimens at room temperature in accordance with the ISO 6892-1 standard [22]. Testing was carried out using a 250 kN Instron 8802 servo-hydraulic machine (Instron, Norwood, MA, USA) operating in position control mode at a crosshead speed of 1 mm/min.

### 3.3. Fractography, Microscopy and EBSD Analysis

Fractured surfaces from selected specimens were examined using a Nikon SMZ 1500 stereomicroscope (Nikon Corporation, Tokyo, Japan). Additional high-magnification observations, both at the centre and near the circumference, were carried out with a JEOL IT-800 HL Scanning Electron Microscope (SEM) (JEOL Ltd., Tokyo, Japan) operating at 20 kV accelerating voltage. Digital Surf analysis conducted by Mountains^®^ 8 image and surface analysis SMILE VIEW™ Map Software [23] (JEOL Ltd., Tokyo, Japan).

The fracture surfaces were observed from a top view with a Nikon SMZ 1500 stereomicroscope. The samples were metallographically prepared and examined, parallel to the longitudinal axis of the tensile sample. The specimens were cold mounted for avoiding annealing effects and metallographically prepared by water grinding with SiC papers and polishing using diamond and silica suspensions.

The observation was performed using a Nikon Epiphot 300 inverted metallographic microscope (Nikon Corporation, Tokyo, Japan) in the as-polished condition. Electron backscatter diffraction (EBSD) analysis was performed using a FEI XL40 SFEG Scanning Electron Microscope (SEM) (FEI Company, Hillsboro, OR, USA) and a JEOL IT-800 HL Scanning Electron Microscope (SEM) under 20 kV accelerating voltage, coupled with an EDAX Apollo XF equivalent to Octane Super EDS and an EDAX Octane Elect Plus (EDAX, Ametek Inc., Obispo, CA, USA), respectively, silicon drift detector (SDD) in cooperation with TEAM software [24] (EDAX, Ametek Inc., Obispo, CA, USA) and an EBSD high-speed camera (EDAX, Ametek Inc., Obispo, CA, USA). EBSD scans were collected using a hexagonal grid. The SEM conditions for the scans were magnification of 200–3000×, accelerating voltage of 20 kV at working distance of 10 mm. Separate grains were considered those with misorientation angle > 5°. Several step sizes were examined, ranging from a minimum step size of 20 nm to maximum step size of 700 nm, in accordance with the selected magnification as well as the metallurgical state of the examined sample.

The post-processing analysis generates Inverse Pole Figure (IPF) maps and plots that display the orientations present, Pole Figure (PF) plots, and misorientation angle grain boundary maps. These maps identify Sub-Grain Boundaries (2–5°, SGBs), Low-Angle Grain Boundaries (5–15°, LAGBs), and High-Angle Grain Boundaries (15–180°, HAGBs). The mean grain size is determined by calculating the mean diameter of each grain.

The kernel average misorientation (KAM) map and diagram are used to illustrate and measure the local grain misorientation and could be corelated mainly with changes due to geometrically necessary dislocations. Black points indicate areas with a low confidence index (<0.05) and therefore they have been excluded from the measurements in order not to alter the calculations. The current EBSD phase library could not identify the phases present within the areas of the retrieved scans.

### 3.4. Thermodynamic Simulation

Calculation of phase diagrams for both minimum and maximum compositions from Table 1 has been conducted with Thermo–Calc^®^ software version 2023b and thermodynamic database for aluminium alloys TCAL8 [25]. The phases used for pseudo–binary phase diagrams are LIQUID, FCC_A1 (Al–matrix), DIAMOND_A4 (Si), MG2SI_C1, AL15SI2M4, AL3TI_LT, AL9FE2SI2.

Moreover, diagrams showing the fraction of precipitation in conjunction with temperature have been extracted from Thermo–Calc^®^ software. Pseudo–binary phase diagrams for minimum and maximum chemical composition of AlSi10Mg were calculated with respect to Si content and maintaining stability of the rest of alloying elements. In addition, a Scheil–Gulliver model with solute solution trapping was employed to describe the solidification path under rapid solidification conditions. Scheil–Gulliver models near infinite cooling rates, in which no diffusion occurs in solid phases whereas the liquid has a homogeneous composition. At the interface of liquid/solid, local equilibrium conditions are always established during solidification [26,27]. Solute trapping in the primary phase (FCC: Al matrix) was considered in the Scheil–Gulliver model for solidification simulation. Scheil–Gulliver with solute trapping is advised to be used in additive manufacturing processes due to the extremely high solidification rates. In additive manufacturing during solidification the interface solid/liquid moves very fast such that even diffusion of alloying elements in liquid phase cannot be completed in the provided time. Hence, alloying elements are trapped near the interface of solid/liquid thus changing the local equilibrium. Increase of solute elements near the interface of solid/liquid is expected to suppress liquidus temperature and impede formation of secondary phases.

## 4. Results and Discussion

### 4.1. Tensile Properties—Experimental Testing

**Figure 4:**
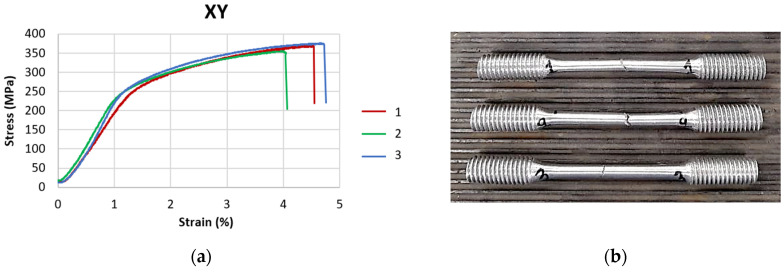
(**a**) The stress-strain dependences recorded for the samples printed in XY direction; (**b**) broken specimens after tensile tests.

Measured experimental data were recorded, processed and evaluated. An example of stress–strain curves for three specimens printed in the XY direction, as well as broken pieces, are shown in Figure 4.

The plots of stress-strain curves for samples printed in other directions and broken specimens, along with fractographic analyses, are given in Appendix A of the manuscript.

**Figure 5:**
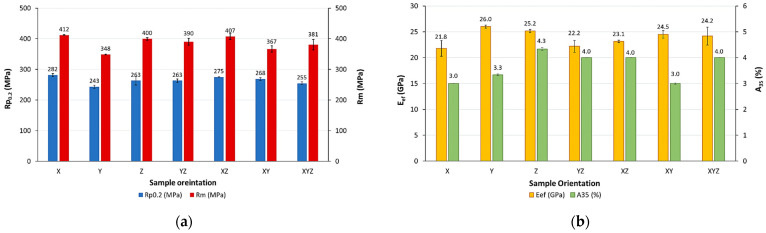
Histograms showing the average values of (**a**) yield strength (R_p0.2_, MPa) and ultimate tensile strength (R_m_, MPa); (**b**) effective elastic modulus (E_ef_, GPa) and elongation (A_35_, %) for each sample orientation group.

For each group of sample orientations, the average value of the mechanical properties was specified, with several records being excluded from further processing (samples X2; YZ3 due to early fracture and XYZ3 due to incorrect thread production). The histograms showing the average values with standard deviation of yield strength (R_p0.2_, MPa), tensile strength (R_m_, MPa), elongation (A_35_, %) and elastic modulus (E, GPa) for each sample orientation group are shown in Figure 5.

The average values of the tensile parameters varied within the following ranges:

- Yield strength (R_p0.2_): from 243 MPa for Y-samples up to 282 MPa for X-samples;
- Ultimate tensile strength (R_m_): from 348 MPa for Y-samples up to 412 MPa for X-samples;
- Elongation (A_35_): 3% for X- and XY-samples up to—4.3% for Z-samples;
- Effective elastic modulus (E_ef_) from 21.8 for X-samples to 26 GPa for Y-samples.

The measured lowest and highest stress values for various orientations of specimens within a 3D-printer workspace showed differences in the range of approximately 15%, which is not a negligible difference. The Y-sample series exhibited the lowest tensile properties, including both yield strength and ultimate tensile strength, as well as the highest elastic modulus. In contrast, the X-sample series demonstrated superior tensile properties, slightly higher than Z- and XZ-groups but minimum effective elastic modulus. The measured elongations A_35_ (%) were quite low (i.e., 3–4.3%), signifying the limited/low ductility of the test pieces, while the highest A_35_ elongation was identified in the Z-series samples. The results also revealed that the Z-series specimens were the most capable of withstanding loads and elastic deformations without failure. If the components were manufactured under the given conditions, this would cause significant limitations for structural applications where toughness is critical.

Based on the experimentally obtained data, it can also be seen that the yield strength, ultimate tensile strength and elongation at break correspond to the values stated in the data sheet [20] (listed in Table 2), but elastic modulus does not. It is almost three times lower than that stated in the data sheet (70 ± 5 GPa); that is a large difference.

In general, there are several factors that affect the elastic modulus of components manufactured using the L-PBF technique. If the particles are too large (close to 80 µm) it could lead to porosity and incomplete melting of the particles; on the other hand, finer powders are more reactive but can more easily agglomerate or accumulate oxides [28,29,30]. The sample manufacturer confirmed by its own analysis a balanced Gaussian distribution of particle sizes with a peak at a particle diameter of 60 µm.

One of the reasons for the decrease in elastic modulus in this case could be the inappropriate choice of energy density to ensure complete fusion of the particles. This is a combination of laser power, scan speed and layer thickness. At a laser power of 500 W, a scanning speed of 2.5 m/s, a spot size of 70 µm and a hatching pitch of 150 µm, the higher scanning speed used could have shortened the residence time in the melt, which could have led to poor layer bonding [31], while a hatching pitch of 150 µm could have left too much space between individual laser passes, which might have resulted in less melt coverage [32,33].

The layer thickness of 30 µm in 3D printing was selected according to standard procedures for fine details and high resolution, but it could have happened that the underlying layers could have had deteriorated conditions for absorbing sufficient heat, which could also have led to poor bonding between layers due to the low thermal gradient [34,35]. The heat treatment to remove residual stresses was carried out based on the recommendation of the standard, which, however, for the selected choice of parameters may not have sufficiently relieved internal stresses or improved the microstructure enough to achieve the full potential modulus of elasticity [36].

The designated stress–relief heat treatment was selected according to ASTM F3318-18 Condition SR1–Parts stress relieved parts [37] which also covers stress–relieving LPBF AlSi10Mg parts. The removal of residual stress was not examined by EBSD analysis as it was not within the scope of our study. In general, anisotropy in the microstructure is not expected to change by removal of residual stresses. Reduction of dislocation density in the microstructure is expected to occur. In order to change the anisotropy in the microstructure, the heat treatment must induce recrystallization and/or precipitation (aging) in the microstructure. Medrano et al., 2023 [38] conducted several heat treatments in an LPBF AlSi7Mg according to ASTM F3318-18, among them a stress–relief heat treatment at 285 °C (±14 °C) for 120 min (±15 min) and subsequently air cooled, which is similar to the stress–relief heat treatment used in our samples. The results of microstructure did not present significant changes compared to the microstructure of non-heat-treated samples. So change was not expected in the microstructure regarding grain size and precipitation.

In Zhang [39], a theoretical model was developed to analyze circular structures produced by selective laser melting, enabling predictions of their geometric accuracy. When employing the 90° rotation scanning strategy, the surface morphology of the circular structures exhibited a series of steps of varying sizes, indicating poor dimensional homogeneity. In contrast, the 67° rotation scanning strategy resulted in nearly uniform dimensional distribution, but the surface remained uneven with dot-like protrusions forming at interlayers. The “skin-core” scanning strategies, utilizing either 90° or 67° rotation for the “core,” produced circular structures with consistent surface morphologies and dimensions. These surfaces displayed subtle, uniform steps and demonstrated good homogeneity in dimensional distribution.

Since the proposed research was not primarily focused on testing the effect of parameters on the elastic modulus, non-standard test conditions compared to ASTM E111 (tensile) [40], or E1875 (ultrasonic) [41] modulus measurement standards could have greatly influenced the measured elastic modulus. The differences in the elastic modulus were only detected after the results were evaluated, therefore for the rest of this manuscript the elastic modulus is not referred to as Young’s modulus, but only as the effective elastic modulus. To determine the root causes of the different tensile behaviour of 3D printed samples to failure and their different mechanical properties, further analyses were performed.

### 4.2. Stereo- and SEM Fractography

Fractographic and topographic observations showing also indicative profilometric aspects are presented in Figure 6, Figure 7, Figure 8, Figure 9, Figure 10, Figure 11, Figure 12, Figure 13, Figure 14, Figure 15, Figure 16 and Figure 17. For comparison purposes, SEM micrographs under ×35, ×200, ×1000 and ×5000 magnification were utilized. Fractography evaluation of the rest of samples is provided in Appendix A.

A detailed examination of the fracture surfaces revealed the presence of multiple fracture mechanisms, exhibiting consistent features across the tested samples. The predominant failure mode was identified as a mixed-mode fracture, combining both ductile and brittle characteristics. This mode was mainly characterized by the coexistence of fine dimpled areas—indicative of micro-void coalescence—alongside features typically associated with limited ductility, such as quasi-cleavage regions and flat, faceted surfaces.

The morphology of the fracture surfaces suggested complex interactions during crack initiation and propagation, possibly influenced by local variations in material properties, residual stresses, and processing parameters. Key fractographic features observed throughout the specimens include:

A. Regions densely populated with fine and shallow dimples, typically formed due to micro-void nucleation and growth under tensile stress, suggesting localized ductile deformation.
B. Tear ridges and interconnected cavities or pores, often aligned along the crack propagation direction, indicative of plastic deformation and the interaction between adjacent micro-voids.
C. Flat facets with mirror-like appearance, commonly associated with quasi-cleavage fracture, reflecting areas of brittle failure possibly initiated at second-phase particles or microstructural inhomogeneities.
D. Un-melted spherical particles of the feedstock material embedded or partially fused at the fracture interface, which may act as stress concentrators or crack initiation sites due to incomplete fusion during processing.

There are several factors that influence the existence of unmelted particles. Technological parameters and settings, including the energy of the laser, greatly affect the energetic conditions of melting metal powder particles and their interactions. Therefore, these technological parameters play a very important role not only in the final quality of the samples, but also in their mechanical properties.

Another key factor for unmelted particles may be that metal powders (due to their cost) are filtered and partially reused after use. Optimal results are achieved when the powder exhibits spherical, smooth particles with a consistent size distribution and minimal contamination. Such properties promote excellent flowability, packing density, and melting performance during the additive manufacturing process. When the powder is repeatedly used, physical and chemical changes may occur that affect its properties. For example, some powder particles may partially melt, sinter, or oxidize due to exposure to high temperatures and reactive gases in the work chamber. These processes can lead to changes such as irregular particle shapes, rough surfaces, increased porosity, and altered composition. Such changes can affect the flowability, packing density, and melting properties of the powder, which can lead to defects such as pores, cracks, or inclusions in the resulting 3D printed components.

Overall, the fractographic evidence points to a synergistic failure mechanism, governed by both material ductility and inherent brittleness, potentially exacerbated by microstructural discontinuities and sub-optimal processing conditions. A detailed analysis is provided in Section 4.2.1, Section 4.2.2 and Section 4.2.3 for samples printed in X, Y and Z direction, respectively.

#### 4.2.1. Sample Printed in X-Direction

**Figure 6:**
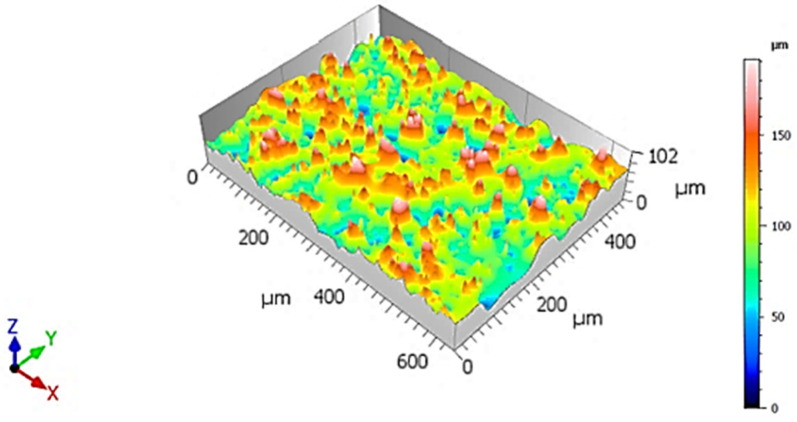
3D visualization of fracture surface of sample X, low-ductility zone adjacent to the circumference.

**Figure 7:**
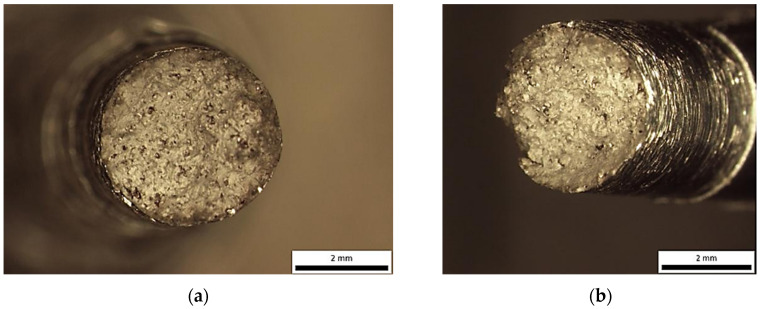
Sample printed in X-direction—Optical stereomicrographs showing the fracture surface (**a**) top view; (**b**) side view.

Topographically, the fracture surface was marked by significant roughness, with multiple elevations and depressions distributed unevenly across the examined area (Figure 6). This pronounced surface relief is indicative of a non-uniform crack propagation front, which did not progress along a flat, planar surface (Figure 7).

**Figure 8:**
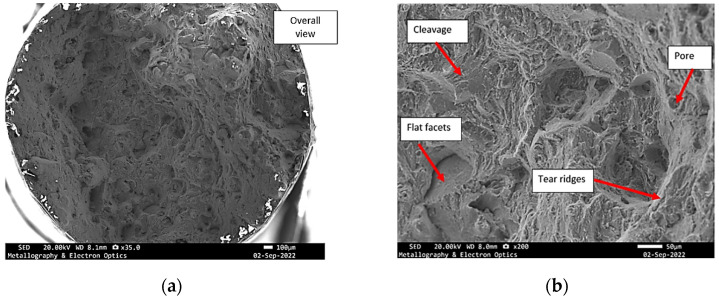
Specimen X—SEM fractographs of specimen X, (**a**) overall view of the fracture surface; (**b**) mixed mode fracture.

**Figure 9:**
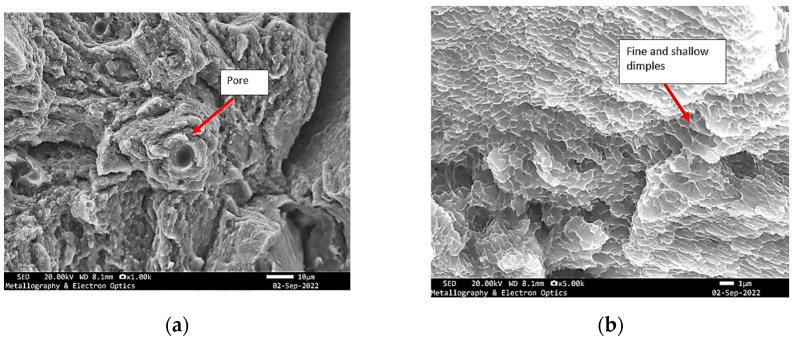
Specimen X—SEM fractographs of specimen X showing (**a**) pores and tear ridges; (**b**) fine and shallow dimples.

Instead, the fracture appears to have developed along an inclined or undulating fracture plane, suggesting that the crack deviated dynamically in response to microstructural heterogeneities, local stress gradients, or the presence of internal flaws. Such morphological characteristics reflect the complex interplay between competing failure modes and highlight the influence of material inhomogeneities and processing-induced defects on crack initiation and propagation. Further examination in SEM shows that the fracture surface exhibited characteristics of a mixed-mode failure, involving both ductile and brittle fracture mechanisms. The presence of discrete pores and cleavage-like regions suggests localized brittle behaviour, while the simultaneous occurrence of fine and shallow dimples indicates areas where ductile micro-void coalescence dominated the failure process (Figure 8 and Figure 9).

#### 4.2.2. Sample Printed in Y-Direction

The fracture surface under examination of the Y-series samples presented secondary fractographic features, which appeared in conjunction with primary ductile and brittle fracture indicators. Specifically, dimples, cavities, flat facets, and pores were all observed, reinforcing the presence of a mixed-mode failure mechanism.

**Figure 10:**
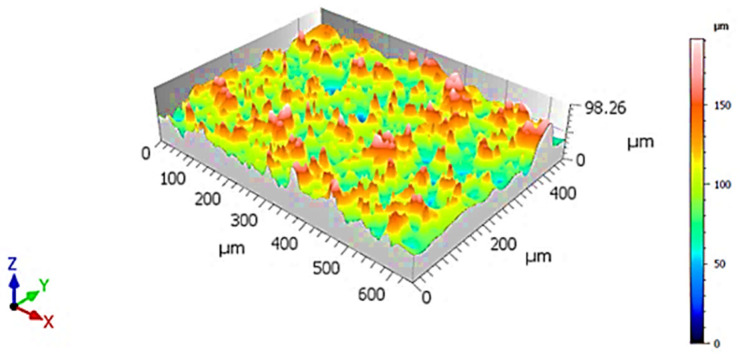
3D visualization of a fracture surface of Y-specimen, ductile zone adjacent to the circumference.

**Figure 11:**
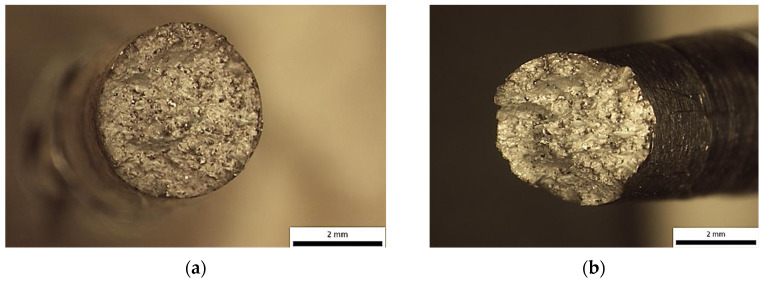
Specimen Y—Optical stereomicrographs showing the fracture surface: (**a**) top view; (**b**) top view.

The general topography of the surface mirrored that of the previous specimen, showing elevated roughness and varying height levels, rather than a uniform, planar separation (Figure 10). This observation suggests a non-parallel fracture propagation path, potentially resulting from heterogeneous microstructural resistance, local stress redistributions, or interactions with internal defects (Figure 11).

**Figure 12:**
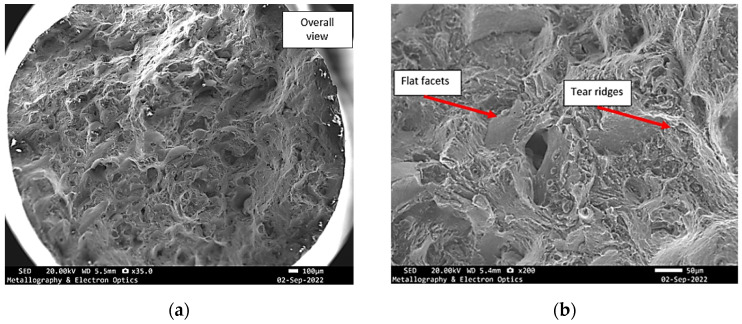
SEM fractography of specimen Y, (**a**) overall view of the fracture surface; (**b**) mixed mode fracture.

Prominent flat facets were also observed, many of which bore characteristics consistent with quasi-cleavage fracture, exhibiting smooth, planar areas with occasional arrest lines. These zones likely represent brittle crack propagation along preferential crystallographic planes, especially in regions of low ductility. Of particular significance was the presence of tear ridges, typically emerging in transition areas between ductile and brittle regions (Figure 12). These morphological features, formed due to localized shear and plastic instability during crack advancement, underscore the non-uniform nature of the failure and further support the classification of the fracture as mixed-mode.

**Figure 13:**
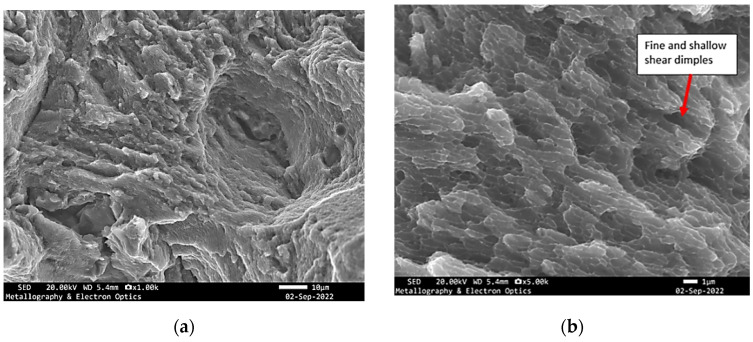
SEM fractography of specimen Y showing (**a**) tear ridges, cavities; (**b**) dimples.

These features closely resembled those identified on the previously analysed fracture surface, indicating similar crack propagation conditions and failure behaviour. The dimples, albeit fine and shallow, were scattered across regions of localized plastic deformation. These were frequently interrupted by cavities and isolated pores, suggestive of internal material discontinuities or process-induced imperfections that may have acted as crack initiation sites (Figure 13).

#### 4.2.3. Sample Printed in Z-Direction

**Figure 14:**
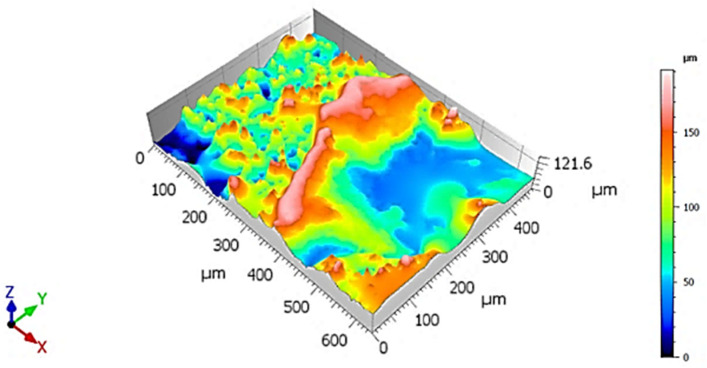
3D visualization of fracture surface of specimen Z, low-ductility zone adjacent to the circumference.

**Figure 15:**
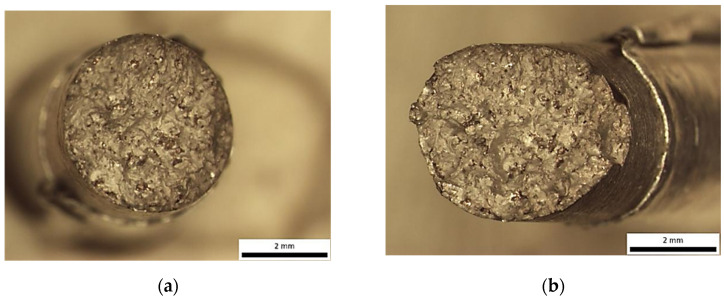
Specimen Z—Optical stereomicrographs showing the fracture surface (**a**) top view; (**b**) side view.

Qualitative assessment of the 3D surface visualization indicated that this area exhibited greater surface depth variation, with larger vertical relief compared to adjacent fracture zones (Figure 14). This observation confirms that the region is topographically deeper, reinforcing the interpretation of intensified localized deformation and energy dissipation during fracture (Figure 15).

**Figure 16:**
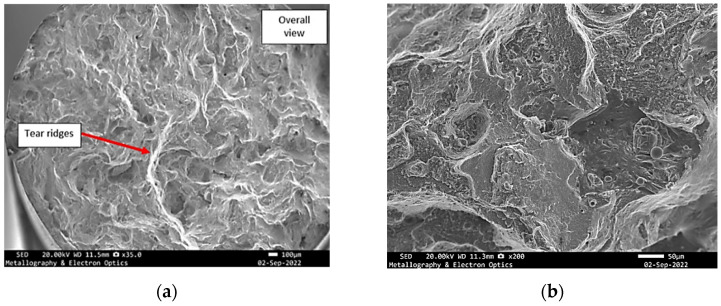
SEM fractography of specimen Z, (**a**) overall view of the fracture surface; (**b**) mixed mode fracture.

A distinct region of the fracture surface was identified, exhibiting a relatively flat morphology interrupted by the presence of well-defined, tall tear ridges. These ridges appeared larger in height and more pronounced compared to surrounding areas, suggesting localized plastic instability during crack propagation. The tear ridges in this zone presented a clear directional alignment, likely following the path of maximum shear deformation (Figure 16).

**Figure 17:**
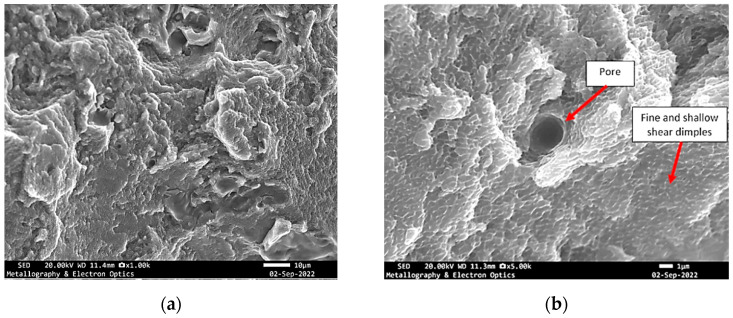
SEM fractography of specimen Z showing (**a**) pores, cavities and tear ridges; (**b**) dimples.

Accompanying features within the same region included fine and shallow dimples, along with discrete cavities and isolated pores, distributed between and around the ridges. These elements point to local ductile behaviour, potentially preceding the more unstable tearing phase (Figure 17).

### 4.3. Optical, Electron Microscopy and Electron Backscatter Diffraction (EBSD) Textural Analysis

#### 4.3.1. Microstructure (Grain Morphology)

The mechanical strength of the samples is strongly influenced by the crystallographic orientation of the grains, their mean size, and morphological characteristics, which are governed by the thermal gradients and solidification conditions during the additive manufacturing process. In laser powder bed fusion (LPBF) specifically, the directionally solidified microstructure tends to develop columnar grains aligned preferentially along the build direction, leading to anisotropic mechanical properties.

In this context, sample Y(3) demonstrated the lowest tensile performance, both in terms of proof stress (R_p0.2_) and ultimate tensile strength (R_m_). This reduction in mechanical response may be attributed to a less favourable grain orientation relative to the loading axis, a coarser or more irregular grain morphology, and possibly a lower texture intensity, which limits the material’s ability to effectively resist deformation and delay crack initiation under tensile load.

On the other hand, sample Z(3) exhibited the highest strength values, indicating a microstructure with grains more optimally oriented along load-bearing directions, potentially finer in size and more uniformly distributed. Such a configuration enhances the material’s capacity to accommodate plastic deformation homogeneously, resulting in higher yield and tensile strength.

Therefore, the comparative mechanical behaviour of the two samples underscores the critical role of grain orientation, size, and morphology, which are intimately linked to the processing parameters, scan strategy, and build orientation employed during fabrication. Subtle differences in these microstructural attributes can lead to pronounced variations in the mechanical performance of AlSi10Mg components manufactured via additive methods.

Multiple studies [42,43,44,45,46,47,48] have examined the effects of different process parameters on the quality of parts produced by SLM technology. These parameters include laser power, scan speed, hatching strategy, layer thickness, and others. According to research by Gibson I. et al. [49], the process parameters affecting porosity in AlSi10Mg SLM components are grouped into four categories:

I. Laser-related parameters—laser power, spot size, pulse duration, pulse frequency;
II. Scan-related parameters—scan speed, scan spacing, scan pattern;
III. Powder-related parameters—particle size, particle shape and distribution, powder bed density, layer thickness, material properties; and
IV. Temperature-related parameters—powder bed temperature, powder feeder temperature, temperature uniformity.

Despite the wide range and variation of technological parameters, these studies report very low porosity (high relative density) of SLM printed samples in the range of 1–3%, but under well-adjusted conditions it is possible to achieve around 0.1%.

**Figure 18:**
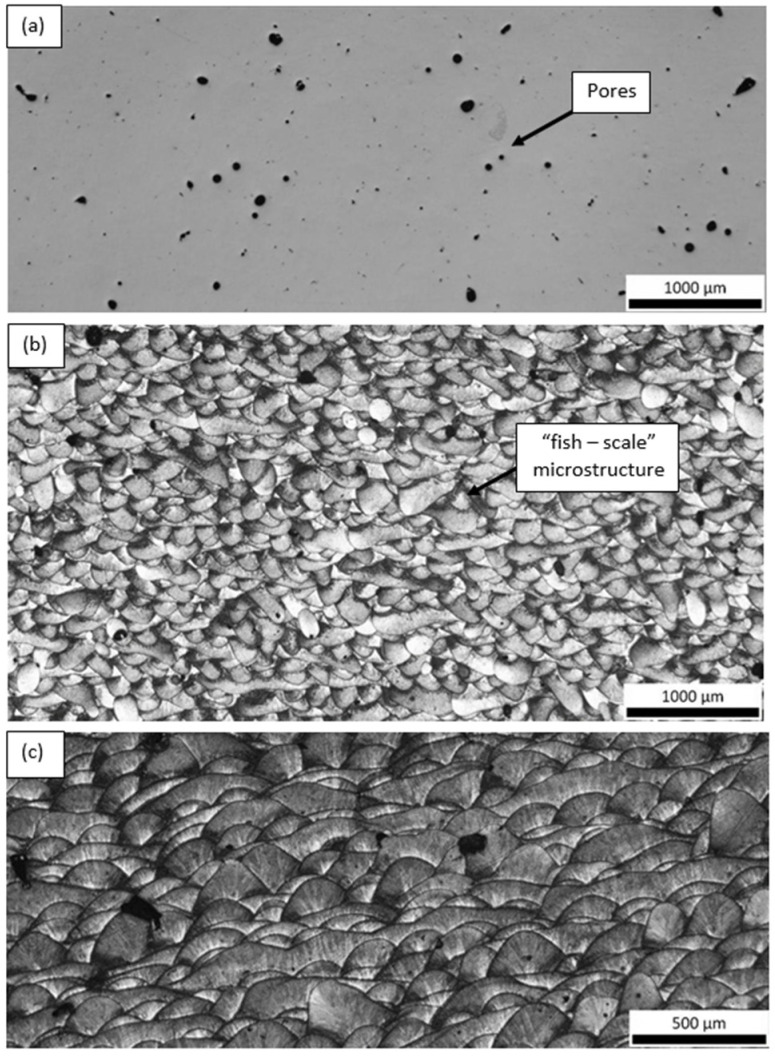
Optical micrographs of the Y(2) sample (**a**) before and (**b**,**c**) after Barker’s etching.

**Figure 19:**
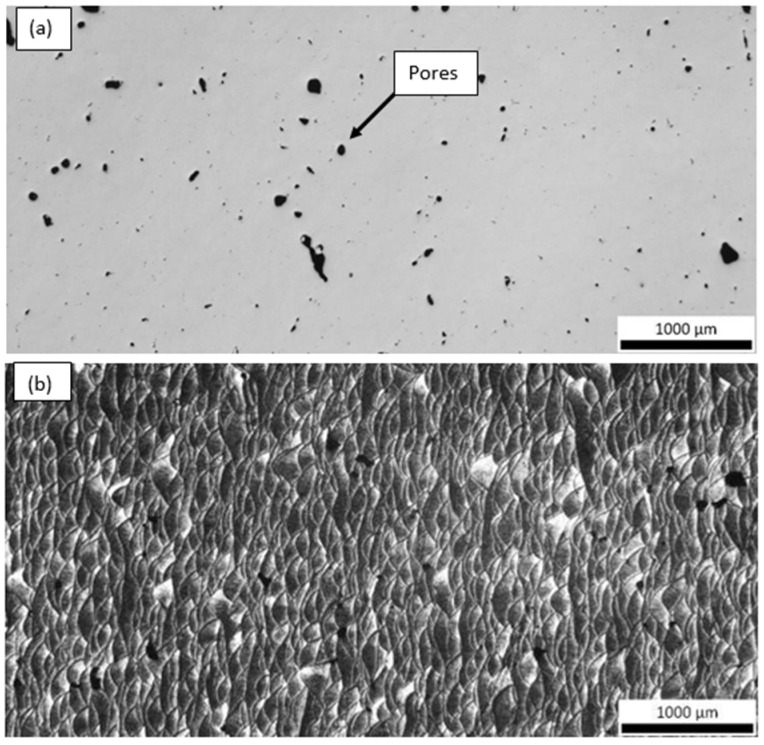
Optical micrographs of the Z(3) sample (**a**) before and (**b**) after Barker’s etching.

Optical microscopy examination of the as-polished condition revealed that both samples contained a high density of pores, distributed throughout the observed cross-sections (Figure 18 and Figure 19). These pores likely originate from incomplete melting, gas entrapment, or lack of fusion during the additive manufacturing process and are known to act as stress concentrators, potentially compromising the mechanical performance of the final component.

In addition to porosity, both samples exhibited a characteristic “fish-scale” microstructure, which is typical of layer-by-layer deposition in LPBF processes. This pattern results from the overlapping melt pools formed during successive laser scans. The curvature and orientation of these melt pool boundaries are highly dependent on the build direction, laser scan strategy, and heat dissipation paths.

Interestingly, the fish-scale morphology was observed from different orientations in the two samples (Figure 18b and Figure 19b), reflecting the influence of build orientation (e.g., Y vs. Z) and sectioning direction on the appearance of the melt pool geometry. This variation offers insight into the thermal history and solidification front progression in each sample, which in turn affects grain growth direction, texture development, and potentially mechanical anisotropy.

The presence of this microstructural feature is not only visually indicative of the additive manufacturing route but also significant from a metallurgical standpoint, as the melt pool boundaries can serve as preferential sites for crack initiation under mechanical loading, especially when aligned perpendicular to the loading axis.

#### 4.3.2. EBSD Analysis

Selective Laser Melting (SLM) does not entail mechanical processing that produces deformation textures; however, the process’s inherent thermal gradients and rapid cooling rates facilitate the epitaxial growth of columnar grains in most aluminum alloys. This preferred grain orientation introduces mechanical anisotropy, influencing properties such as yield strength and elongation at failure, while also increasing susceptibility to cracking. The crystallographic texture found in SLM-fabricated aluminum components results from the directional solidification within the melt pool. Both the morphology of the melt pool and the direction of heat flow at the liquid/solid interface, alongside the solidification rate, are predominantly governed by processing parameters and the material’s thermo-physical characteristics. Consequently, the strength of the resulting grain texture can differ substantially depending on the equipment and feedstock employed [50].

In the early phases of melt pool solidification, the predominant grain growth orientation is governed by the direction of the solidification front—generally perpendicular to the melt pool boundary—and by prevailing thermal gradients. These thermal gradients are primarily oriented either opposite to the build direction or radially, contingent upon the melt pool’s width-to-depth ratio. This phenomenon results from the limited constitutional undercooling found in most aluminum alloys, attributable to their high thermal conductivity and rapid solidification rates [51]. Consequently, a distinctive morphological grain texture develops, with the longitudinal cross-section of SLM components revealing elongated grains that originate at the boundaries of the melt pool.

**Figure 20:**
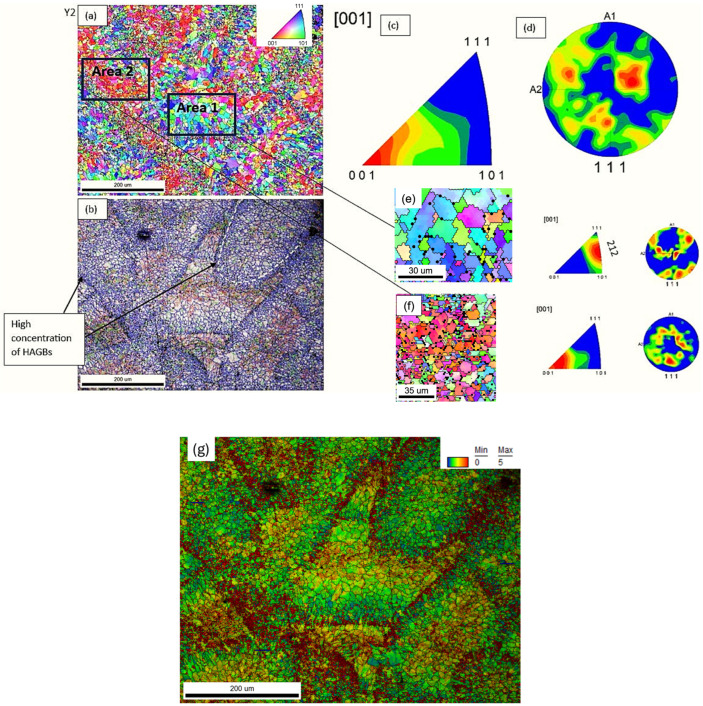
(**a**) IPF map, (**b**) misorientation angle grain boundaries map, (**c**) IPF plot, (**d**) PF plot of sample Y(3) and (**e**) IPF map, plot and PF plot of sample Y(3)—Area 1, (**f**) IPF map, plot and PF plot of sample Y(3)—Area 2; (**g**) KAM map of the same area as area from maps (**a**,**b**).

This kind of grain morphology is also linked to a crystallographic texture in metals that display “easy growth” directions. For example, in aluminum alloys, dendrites whose 〈100〉 axes are oriented along the solidification front will suppress grains that are not as well aligned (see Figure 20).

Wu et al. [52] demonstrated that elongated grains positioned near the melt pool boundaries in SLM-processed AlSi10Mg consist of sub-cells with consistent orientation alignment. Thijs et al. [53] additionally determined that this prevailing grain orientation is responsible for the formation of a 〈100〉 fiber texture component along the scan direction. Comparable findings were presented by Suryawanshi et al. [54] for AlSi12 alloys. Conversely, Takata et al. [55], Zhang et al. [56], and reference [44] provided evidence that the elongated grain structures in AlSi10Mg and Al-Mg-Cu exhibit a dominant 〈100〉 texture along the build direction. These observed discrepancies can be attributed to variations in melt pool geometry and the associated thermal gradients. During solidification, elongated grains may assimilate remaining liquid [51,57], or alternatively, a refined equiaxed grain structure can develop. Equiaxed grains, characterized by the absence of a preferred crystallographic texture, are generally desirable due to their ability to mitigate mechanical anisotropy. Current research focuses on optimizing melt pool structure to minimize crystallographic texture and reduce the incidence of solidification cracking [51,56,58,59,60,61,62].

The way crystallographic texture develops in SLM-produced components depends on how individual melt pools and tracks combine. Factors like partial re-melting of adjacent tracks can affect the intensity of this texture. When partial re-melting occurs, the initially formed fine equiaxed structure at the end of melt pool solidification can be replaced by longer, textured grains. As a result, even when processing the same alloy, varying levels of texture intensity may emerge, since the amount of partial re-melting in nearby tracks influences the proportion of textured grains. This effect is shaped by parameters such as hatch spacing, layer thickness, and the chosen laser scanning strategy [53,63].

The research which has already been conducted indicates that both inter-layer and intra-layer rotation of the scan strategy are effective approaches to reducing texture. Specifically, for AlSi10Mg, where the movement of the laser typically generates elongated melt pools, rotating the scan direction lessens the extent of re-melted material when compared to uni- or bi-directional strategies. This rotational technique promotes the preservation of randomly oriented equiaxed grain structures established during the initial solidification of the melt pool [53,64].

The EBSD analysis performed in the context of this investigation provided crucial insights into the grain boundary character distribution and crystallographic texture of the two AlSi10Mg samples fabricated by LPBF.

The inverse pole figure (IPF) mapping revealed that sample Y(3) was characterized by two well-defined crystallographic orientation clusters, namely along the <212> and <001> directions (Figure 20a). This pronounced bimodal texture suggests the presence of microstructural domains with different mechanical responses under loading. The stronger texture intensity in the <212> areas, reflected by a pole figure (PF) intensity of 5.467, compared to 3.179 in the <001> region, highlights the potential for differential plastic deformation behaviour within the same sample. Such localized mechanical incompatibility could act as a driver for crack initiation and propagation, particularly under cyclic or tensile loading.

Furthermore, sample Y(3) exhibited a microstructure predominantly composed of high-angle grain boundaries (HAGBs), which accounted for more than 70% of the boundaries, while low-angle grain boundaries (LAGBs) were below 20% (Table 3, Figure 20b). The high fraction of HAGBs typically indicates a more recrystallized microstructure, with reduced dislocation substructure continuity across grain boundaries. This configuration can lead to reduced intergranular compatibility during deformation and promote strain localization along specific boundaries.

**Figure 21:**
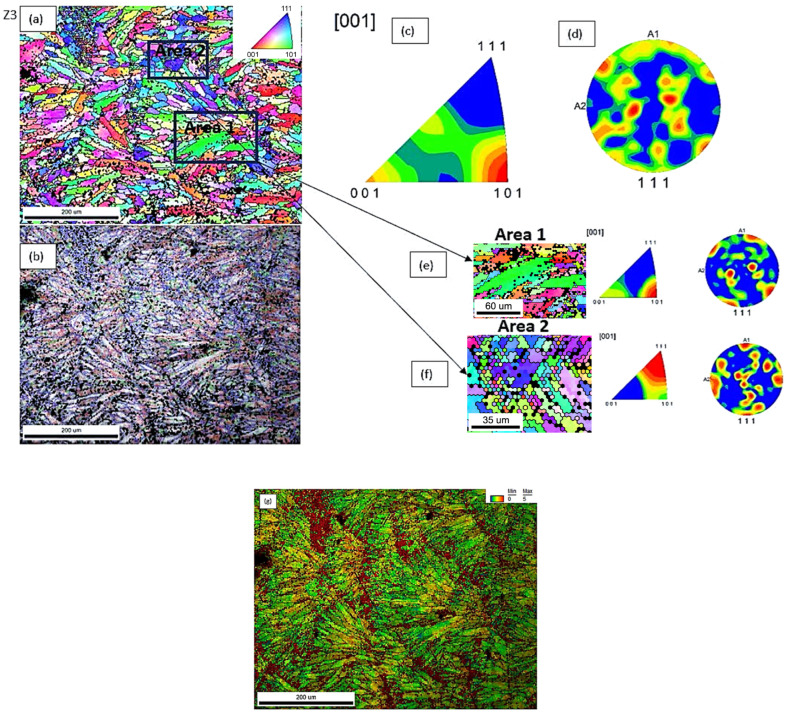
(**a**) IPF map, (**b**) misorientation angle grain boundaries map, (**c**) IPF plot, (**d**) PF plot of sample Z(3), (**e**) IPF map, plot and PF plot of sample Z(3)—Area 1; (**f**) IPF map, plot and PF plot of sample Z(3)—Area 2; (**g**) KAM map of the same area as area from maps (**a**,**b**).

In contrast, sample Z(3) exhibited a more randomized grain orientation distribution, with less pronounced texture and more heterogeneous mixing of grain orientations (Figure 21a). The grain boundary analysis showed a higher fraction of LAGBs (>30%) and a reduced percentage of HAGBs (<60%) (Figure 21b). The elevated LAGB content is often associated with a greater presence of subgrain structures and enhanced dislocation accommodation across boundaries, enabling better grain compatibility and coalescence during plastic deformation. This microstructural characteristic can contribute to improved strain distribution, delaying crack nucleation and thus correlating with the superior mechanical performance observed for this sample. Further insight into the deformation behaviour was provided by the analysis of the Kernel Average Misorientation (KAM) parameter, which reflects the local lattice curvature associated with geometrically necessary dislocations (GNDs) and serves as a qualitative indicator of residual plastic strain and local strain gradients. The overall KAM value was found to be higher in sample Z(3) (2.67953) compared to sample Y(3) (2.48951), indicating a higher density of local misorientations in the former. This suggests that sample Z(3) experienced more extensive plastic deformation at the microscale, possibly due to its more favourable grain boundary character (higher LAGB content) and random grain orientation, which allows better dislocation mobility and redistribution of stress (Table 3).

However, the KAM gradient, i.e., the variation of misorientation within specific regions, was observed to be higher in sample Y(3) (approximately 0.8) than in sample Z(3) (approximately 0.5) (Figure 20g and Figure 21g). This indicates that sample Y(3), despite its lower overall misorientation, exhibits sharper transitions in lattice orientation between adjacent grains or domains, which could reflect localized deformation bands or regions of stress concentration. Such heterogeneity in plastic strain accommodation may facilitate preferential fracture paths, especially under tensile loading. In contrast, the more uniform KAM distribution in sample Z(3) points toward smoother dislocation flow and better compatibility between grains during deformation, despite the marginally higher residual strain level. This behaviour is consistent with its improved tensile properties and highlights the complex interplay between grain size, texture, misorientation, and strain localization in determining the mechanical performance of LPBF-manufactured alloys.

Similarly, according to a recent study pertaining to the texture analysis of as-drawn and heat-treated brass alloys, the higher fraction of LAGBs and Coincident Site Lattice (CSL) boundaries favour ductile and transgranular cracking, increasing fracture resistance [65].

**Figure 22:**
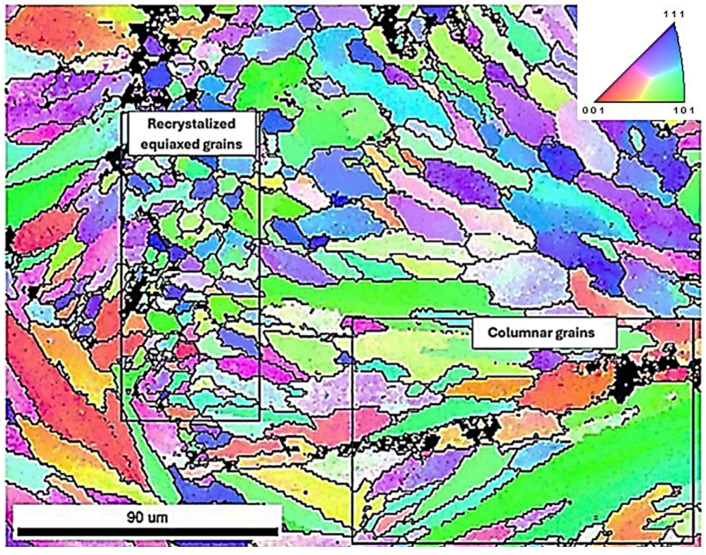
IPF map at higher magnification of sample Z(3) exhibiting the coexistence of fine recrystallized equiaxed grains with columnar grains. Note the random texture of the area examined highlighted by the color coding.

Furthermore, the lower PF and IPF intensities recorded for sample Z(3) reflect a weaker overall texture (Figure 22), suggesting a more isotropic mechanical behaviour, with fewer preferential paths for crack propagation and less likelihood of strain localization.

In summary, the EBSD findings correlate well with the tensile behaviour of the samples: sample Y(3), with its strong texture, high HAGB content, and orientation clustering, presents conditions prone to localized failure, while sample Z(3) benefits from a more balanced, deformable grain network supporting enhanced mechanical integrity.

Quantitative grain size measurements, performed via EBSD analysis, revealed notable differences in the microstructural scale between the two examined AlSi10Mg samples. Specifically, sample Y(3) presented a mean grain size of approximately 5 μm, with localized variations ranging from 5 to 7 μm, while sample Z(3) exhibited larger grains, with size values spanning between 7 and 9 μm (Table 3).

From a classical standpoint, finer grain structures are typically associated with enhanced yield strength, in accordance with the Hall–Petch relationship, which predicts that decreasing grain size leads to increased resistance to dislocation motion. However, this relationship did not appear to hold in the present case: sample Y(3), despite its finer grains, exhibited lower mechanical strength compared to sample Z(3). This apparent deviation can be attributed to the influence of strong crystallographic texture in sample Y(3), which may promote anisotropic deformation and strain localization, effectively counteracting the grain refinement effect [66].

### 4.4. Thermodynamic and Scheil–Gulliver Simulation

**Figure 23:**
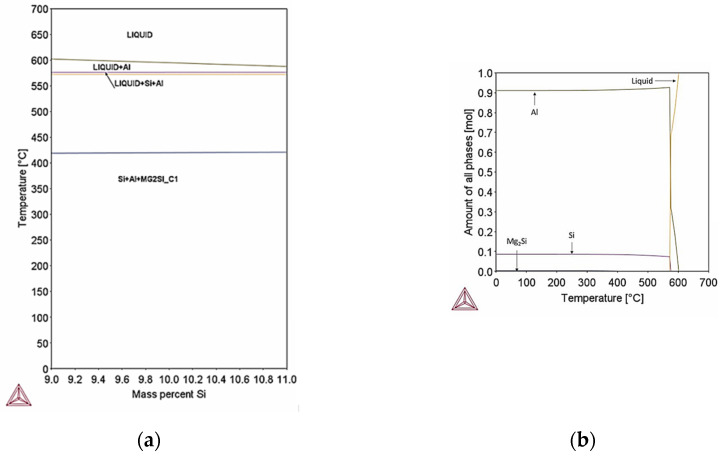
(**a**) Pseudo-binary phase diagram of minimum composition of AlSi10Mg; (**b**) Mass fraction of phases formed in minimum composition of Al10SiMg. Precipitation of Si particles in Al matrix is expected at equilibrium conditions. Mg2Si phase is expected to form below 425 °C; however, the percentage of Mg2Si phase is very low, therefore in extreme cooling conditions it will not appear.

**Figure 24:**
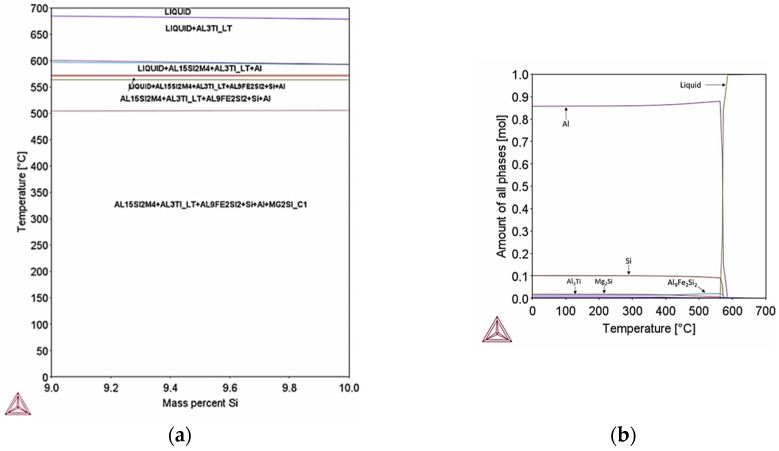
(**a**) Pseudo–binary phase diagram of maximum composition of AlSi10Mg, (**b**) Mass fraction of phases formed in maximum composition of Al10SiMg. Precipitation of Si particles is expected along with Fe rich intermetallic phases like Al15Si2M4 and Al9Fe2Si2 and Mg2Si and Al3Ti. The sum of precipitates apart from Si particles is well below 5% mol percent, therefore at rapid cooling rates their formation is expected to be suppressed.

Figure 23 and Figure 24 show the pseudo–binary phase diagrams for minimum and maximum chemical composition of AlSi10Mg. For minimum chemical composition the Si (9% wt.) phase appears at the end of solidification (Figure 23a). Si phase precipitation in the Al matrix up to 10% mol percent (Figure 23b). The percentage of precipitation is directly related to the process conditions existing during manufacturing.

In maximum chemical composition apart from the Si phase which is expected to precipitate up to 10% mol percent at equilibrium conditions, phases Al15Si2M4 (M: Fe, Mn), Al9Fe2Si2 and Al3Ti are expected to form at temperatures below 552 °C (Figure 24a). The sum of precipitation of Al15Si2M4, Al9Fe2Si2 and Al3Ti is expected to be lower than 5% mol percent (Figure 24b); however, since the additive manufacturing process involves ultra–fast cooling rates (10^3^–10^6^ °C/s) the formation of these phases is expected to be suppressed. Si phase derives from the eutectic reaction L → Al + Si.

As reported in the literature [67], additively manufactured AlSi10Mg components feature a fine network of silicon particles distributed within the aluminum matrix. The mechanical properties are improved through modifications to the eutectic phase. Formation of the fibrous silicon network surrounding the aluminum matrix results from the ejection of silicon particles during rapid cooling. Additionally, the fine grains and dislocations produced by additive manufacturing interact with silicon particles to enhance the strength of AlSi10Mg via mechanisms such as Hall–Petch, Orowan, and dislocation hardening [67].

**Figure 25:**
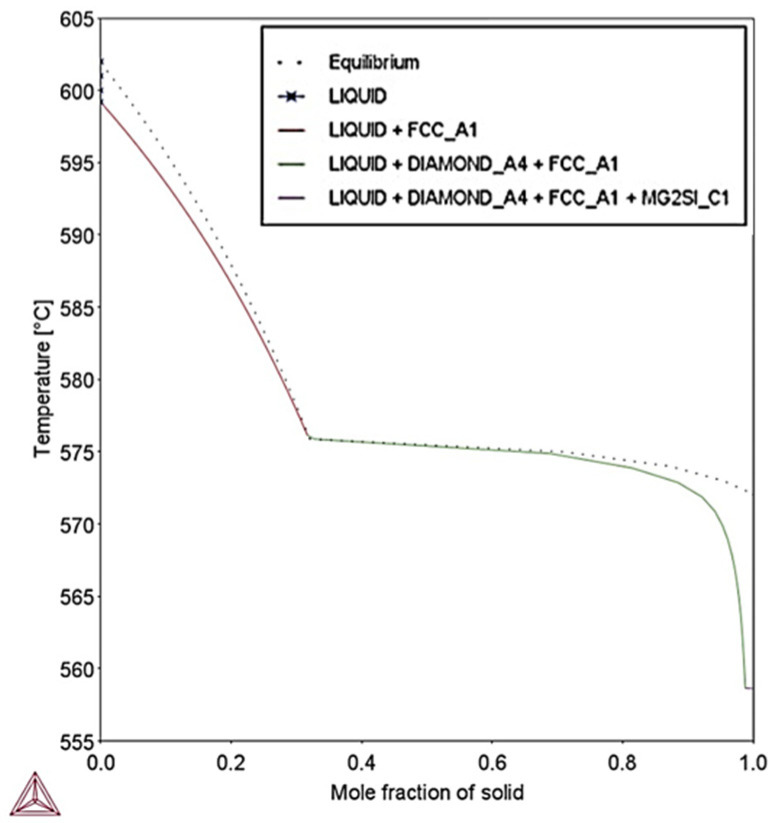
Solidification paths calculated by Scheil–Gulliver and equilibrium conditions for minimum AlSi10Mg composition. Scheil–Gulliver presents the highest freezing range compared to equilibrium conditions.

Due to the extreme cooling conditions that prevail during SLM, the Scheil–Gulliver model has been utilized. Figure 25 shows the solidification paths for minimum AlSi10Mg concentration for equilibrium and Scheil–Gulliver simulations with respect to temperature and fraction of solid. It presents the greatest freezing range of 40.4 °C, whereas the freezing range calculated at equilibrium conditions was 30 °C.

**Figure 26:**
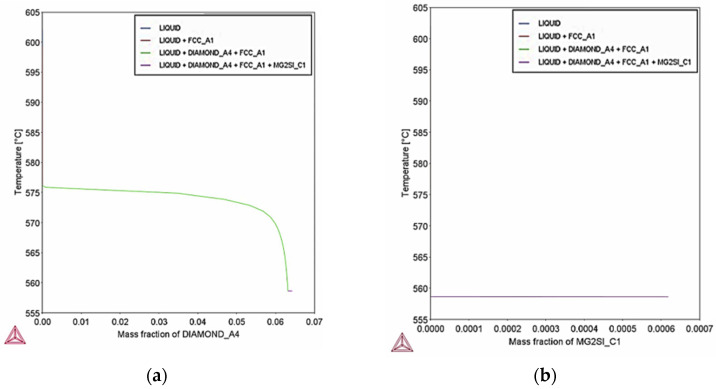
Scheil–Gulliver simulation for minimum composition of AlSi10Mg, (**a**) fraction of Si particles during solidification; (**b**) fraction of Mg2Si phase during solidification. Precipitation of Si particles is expected to reach 6.3% mass percent whereas Mg2Si phase 0.06% mass percent.

Scheil–Gulliver simulation indicates also that at extreme cooling conditions the percent of precipitation of Si particles is 6.3% mass percent (Figure 26a), which is lower than the percent of Si particles calculated at equilibrium. The decrease of Si particles can be attributed to the rapid cooling rate that suppress Si diffusion. The percent of Mg2Si particles is expected to be 0.06% mass percent (Figure 26b) which is slightly higher than equilibrium (0.03% mass percent), probably due to Mg enrichment in liquid.

**Figure 27:**
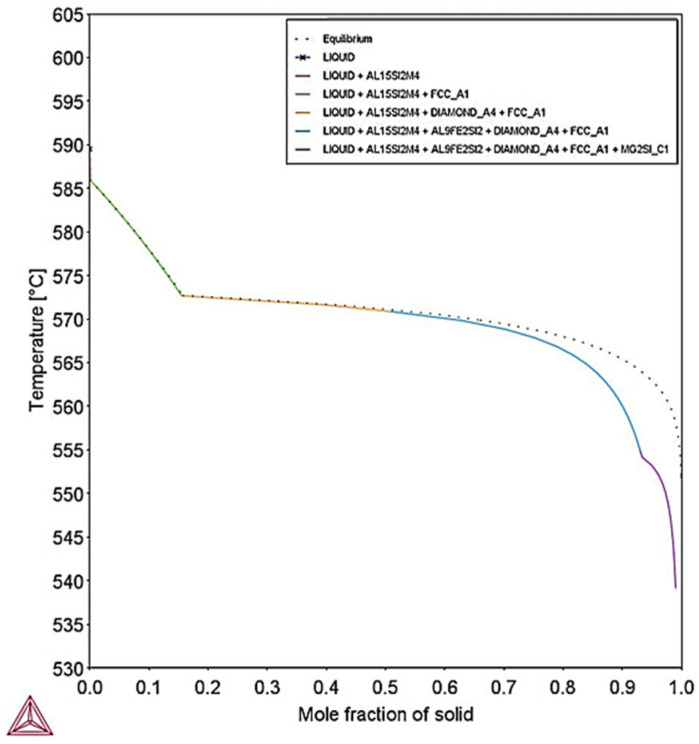
Solidification paths calculated by Scheil–Gulliver and equilibrium conditions for maximum AlSi10Mg composition. Scheil–Gulliver presents the highest freezing range compared to equilibrium conditions.

Figure 27 shows the solidification paths for maximum AlSi10Mg concentration for equilibrium and Scheil–Gulliver simulations with respect to temperature and fraction of solid. Similarly, to minimum composition of AlSi10Mg, Scheil–Gulliver model presents the greatest freezing range of 50.8 °C, whereas the freezing range calculated at equilibrium conditions was 38.3 °C.

**Figure 28:**
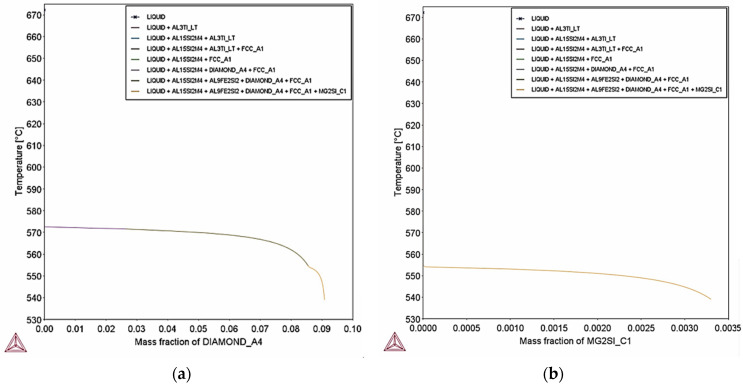
Scheil–Gulliver simulation for maximum composition of AlSi10Mg, (**a**) fraction of Si particles during solidification; (**b**) fraction of Mg2Si phase during solidification. Precipitation of Si particles is expected to reach 9% mass percentage, whereas Mg2Si phase has 0.33% mass percentage.

Scheil–Gulliver simulation indicates also that at extreme cooling conditions Si particles, Mg2Si, Al15Si2M4 (M: Fe, Mn), Al9Fe2Si2 and Al3Ti form. The percent of precipitation of Si particles is 9% mass percentage (Figure 28a) which is lower than the percent of Si particles calculated at equilibrium. The decrease of Si particles can be attributed to the rapid cooling rate that suppresses Si diffusion. The percentage of Mg2Si particles is expected to be 0.33% mass percent (Figure 28b) which is equal to equilibrium (0.3% mass percent).

Thermo–Calc and Scheil–Gulliver simulations were used in order to estimate the expected percent of precipitation that may have formed in the microstructure and the type of intermetallic phases after laser powder bed fusion. Taking into consideration the simulations for minimum and maximum chemical composition, mostly Si particles are expected to form in the microstructure at a percent of 6–9% *w*/*w* which is expected since Si content is max 10% and has very low solubility in Al matrix. Moreover, the rapid cooling suppresses Si diffusion therefore not all Si can precipitate in the matrix. AlSi10Mg parts manufactured by laser powder bed fusion have been studied extensively in literature and the microstructures observed have mostly fine Si particles precipitated in the Al matrix. Phase identification can be performed but it will give further insight into the other phases that are expected to form (e.g., Mg2Si, Al15Si2M4, Al9Fe2Si2 and Al3Ti), which is a significantly smaller percentage than Si particles. The total precipitation of the aforementioned phases is expected to be 2% at equilibrium conditions. Provided the rapid cooling suppresses the diffusion of alloying elements, certain phases expected at equilibrium may not form at all.

The observed ⟨100⟩ texture in our study originates from the same physical mechanism described there: during directional solidification under sharp thermal gradients, columnar grains grow epitaxially along the melt pool boundaries following the heat flow direction. Since aluminum exhibits an FCC lattice where the ⟨100⟩ direction corresponds to the easiest crystal growth path, dendritic structures preferentially align along ⟨100⟩, leading to a strong fiber texture. As reported by Thijs et al., the intensity of this texture depends on the local solidification conditions and scan strategy, fully supporting the texture evolution and anisotropic mechanical response discussed in the present work [68].

## 5. Conclusions

The tensile behaviour of differently oriented 3D-printed AlSi10Mg dog-bone specimens was studied and presented in the frame of this research. Tensile testing (21 specimens in total, covering all the different orientations) was performed until failure on 3D-printed AlSi10Mg dog-bone specimens at ambient temperature, according to ISO 6892-1 international standard. This study determines which of the 3D printing directions of the AlSi10Mg aluminium alloy is the weakest under given conditions and which may be the most likely cause of failure of components containing structures (features) printed in different directions under tensile loading, since the whole is only as strong as its weakest link, which is a very important aspect for design purposes to model and optimize the performance of 3D structures, as well as for failure analysis and, above all, for failure prevention. The investigation was carried out along with the determination of the failure mechanisms and their correlations with the different microstructural and textural properties inherited due to the different 3D printing directions. The main findings are as follows:

- Yield strength (R_p0.2_), ultimate strength (R_m_), elongation (A_35_) elongation at break and Young’s modulus were evaluated.
- The Y-sample series showed the minimum tensile properties (yield 243 MPa and ultimate 348 MPa tensile strength), while the X-sample series showed the maximum ones (282 MPa and 412 MPa). The strength of the samples is highly dependent on the orientation of grains, their size and morphology. Individually evaluated, the Y(3) sample showed the minimum tensile properties (A_35_ = 4%, R_p0.2_ = 239 MPa and R_m_ = 346 MPa), while Z(3) showed the maximum ones (A_35_ = 4%, R_p0.2_ = 273 MPa and R_m_ = 420 MPa). Fractographic investigation on tensile fractures identified predominantly a low ductility failure mechanism, showing fine size shear (mostly), dimple formation, tear ridges, flat facets, pores and cavities as well as spheres of un-melted feed material.
- Sample Y(3) exhibited a strong, dual-component crystallographic texture, with two dominant orientation clusters along the <212> and <001> directions. The disparity in texture intensity between these regions is likely responsible for localized mechanical incompatibilities and strain heterogeneity, resulting in preferential crack paths and reduced mechanical strength. In contrast, sample Z(3) presented a more randomized orientation distribution, with less distinguishable texture zones, enabling better strain accommodation and more uniform plastic deformation, which correlates with its higher tensile and yield strength.
- Although sample Y(3) had finer grains (5–7 μm) compared to Z(3) (7–9 μm), it exhibited lower mechanical strength. This deviation from the Hall–Petch relationship is attributed to the dominant effect of strong crystallographic texture in Y(3), which counteracted the expected strengthening effect of grain refinement. This highlights the complex interplay between grain size and texture in LPBF-produced materials.
- Sample Y(3) contained a higher fraction of high-angle grain boundaries (HAGBs) (>70%) and lower low-angle grain boundary (LAGB) content (<20%), indicative of a more recrystallized but less accommodating microstructure. On the other hand, Z(3), with LAGBs >30%, supported smoother dislocation movement and enhanced grain compatibility, contributing to improved ductility and resistance to fracture.
- Despite Z(3) showing a higher overall KAM value (2.68 vs. 2.48 in Y(3)), its KAM gradient was lower (~0.5) compared to Y(3) (~0.8). This suggests a more homogeneous distribution of local misorientation in Z(3), implying less abrupt strain gradients and lower risk of strain localization, whereas Y(3) may exhibit more concentrated deformation bands prone to crack initiation.
- Both samples demonstrated mixed-mode fracture characteristics, with dimples, cavities, pores, and flat facets. Tear ridges were more prominent and vertically developed in certain regions, especially in areas with flat fracture planes and deeper topographical features (confirmed via 3D visualization). These regions likely indicate localized plastic instability and strain concentration.
- The direction <101> accompanied by the most isotropic microstructure of sample Z(3) as well as the lower KAM gradient affect between the different areas observed led to superior mechanical response in contrast to sample Y(3) although the latter exhibited finer mean grain size.
- The results show that precipitation of Si is expected both on minimum and maximum composition reaching up to 10%. In case of maximum composition, precipitation of other phases is observed at lower percentage (<5%) such as Al15Si2M4, (M:Fe, Mn) Al9Fe2Si2 and Mg2Si.

## 6. Suggestions for Further Research

Based on preliminary research and the results achieved, the authors would like to explore the following topics in greater depth and detail in the near future:

- quantification of porosity, microstructure, texture and defects for a full set of different sample orientations to investigate whether there is any correlation with the observed mechanical behavior;
- expanding EBSD mapping to all orientations to justify anisotropy trends;
- providing a detailed view on each orientation summarizing grain size, texture intensity, porosity, yield/UTS, and elongation, including Hall–Petch together with an anisotropy factor for more physical interpretations.

## Figures and Tables

**Table 1:** Mechanical properties of stress relieved AlSi10Mg alloy [20] in both XY and Z directions (* as build).

Property	Value
Ultimate tensile strength (MPa)	410
Yield strength (MPa)	240
Young’s modulus (GPa)	70 ± 5
Elongation at break * (%)	5 ± 2

**Table 2:** Chemical composition of AlSi10Mg alloy [20].

Element	Al	Mg	Si	Ni	Sn	Pb	Cu	Zn	Ti	Mn	Fe
wt. (%)	bal.	0.2–0.45	9–11	<0.05	<0.05	<0.05	<0.05	<0.1	<0.15	<0.45	<0.55

**Table 3:** Values retrieved by the post-processing of the EBSD scans of samples Y(3) and Z(3).

Sample	Misorientation Angle Grain Boundaries (°)		Main Directions	IPF	PF	Grain Size (Mean Diameter, μm)	KAM	KAM Gradient
Y(3) (all the scanned area)	2–5°	18.4	<001>	1.226	1.437	5.3	2.48951	
	5–15°	11.7						
	15–180°	69.9						
Y(3) (Area 1)	2–5°	21.2	<212>	3.033	5.467	7.1	1.73955	0.8
	5–15°	8.4						
	15–180°	70.4						
Y(3) (Area 2)	2–5°	18.4	<001>	2.583	3.179	5.1	2.50741	
	5–15°	11.7						
	15–180°	69.9						
Z(3) (all the scanned area)	2–5°	37.0	<101> <001>	1.451	1.943	8.7	2.67953	
	5–15°	12.3						
	15–180°	50.7						
Z(3) (Area 1)	2–5°	17.3	<101>	1.561	3.541	7.0	2.22937	0.5
	5–15°	7.4						
	15–180°	75.3						
Z(3) (Area 2)	2–5°	39.9	<111>	4.617	7.528	9.3	2.73511	
	5–15°	13.5						
	15–180°	46.6						

**Figure A1:**
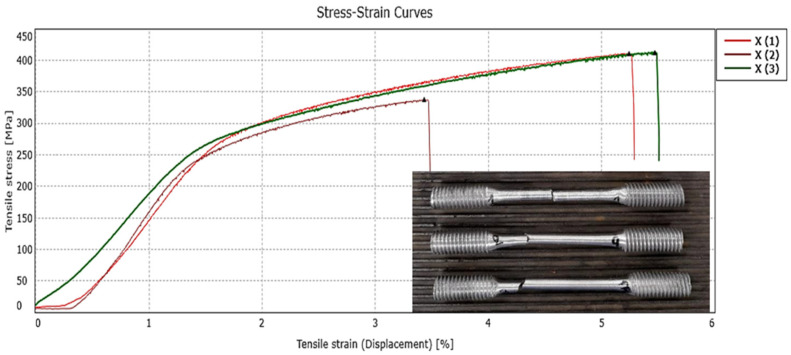
Stress–strain curves obtained after three replicate specimens tensile test (Sample X).

**Figure A2:**
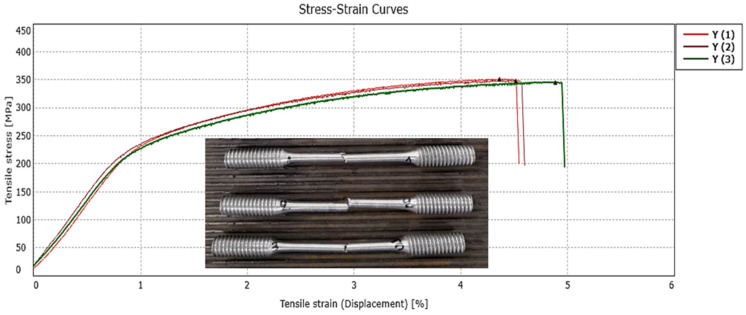
Stress–strain curves obtained after three replicate specimens tensile test (Sample Y).

**Figure A3:**
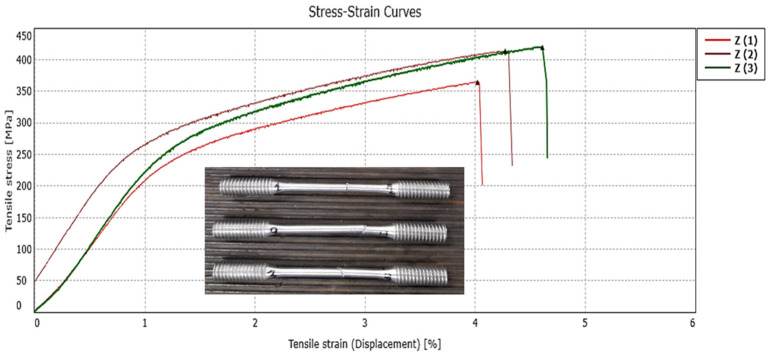
Stress–strain curves obtained after three replicate specimens tensile test (Sample Z).

**Figure A4:**
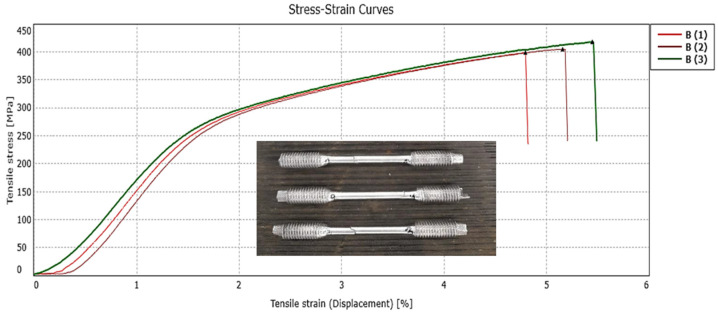
Stress–strain curves obtained after three replicate specimens tensile test (Sample B–XZ).

**Figure A5:**
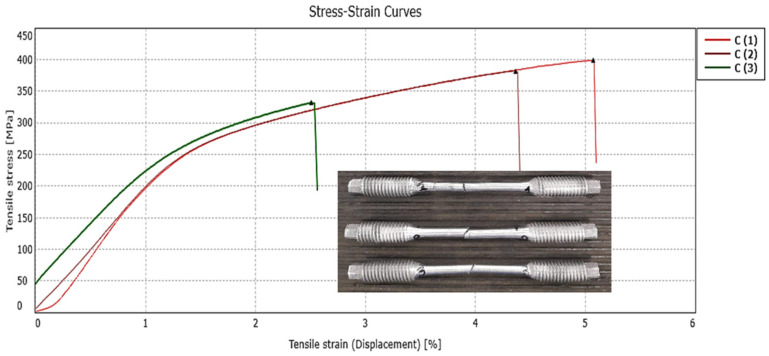
Stress–strain curves obtained after three replicate specimens tensile test (Sample C–YZ).

**Figure A6:**
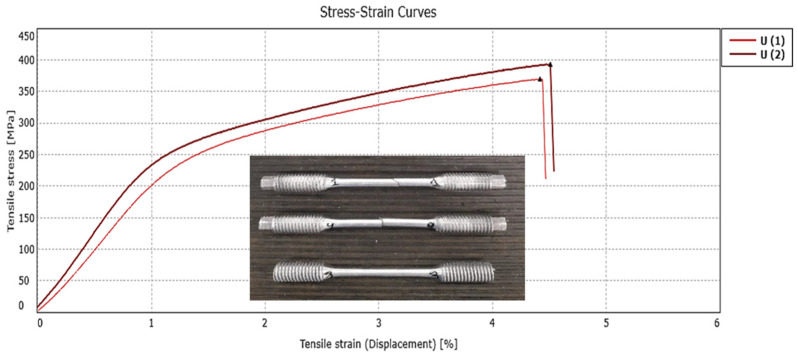
Stress–strain curves obtained after three replicate specimens tensile test (Sample U–XYZ).

## Data Availability

The original contributions presented in this study are included in the article. Further inquiries can be directed to the corresponding authors.

## Appendix A

Results from tensile testing and fractography from the rest of the samples are provided below.

**Table A1:** Tensile test results (Sample X).

No	Sample ID	Diameter (mm)	Max. Load (kN)	Rp0.2 (MPa) *	Rm (MPa)	A_35_ (%)
1	X (1)	3.64	4.3	285	411	3
2	X (2)	3.48	Rejected due to early breakage			
3	X (3)	3.81	4.7	278	413	3
**AVERAGE VALUES**				**282**	**412**	**3**

* Indicative Value.

**Table A2:** Tensile test results (Sample Y).

No	Sample ID	Diameter (mm)	Max. Load (kN)	Rp0.2 (MPa) *	Rm (MPa)	A35 (%)
1	Y (1)	3.63	3.6	249	351	3
2	Y (2)	3.68	3.7	241	348	3
3	Y (3)	3.73	3.8	239	346	4
**AVERAGE VALUES**				**243**	**348**	**3**

* Indicative Value.

**Table A3:** Tensile test results (Sample Z).

No	Sample ID	Diameter (mm)	Max. Load (kN)	Rp0.2 (MPa) *	Rm (MPa)	A35 (%)
1	Z (1)	3.97	4.5	247	365	4
2	Z (2)	3.83	4.8	269	414	5
3	Z (3)	3.77	4.7	273	420	4
**AVERAGE VALUES**				**263**	**400**	**4**

* Indicative Value.

**Table A4:** Tensile test results (Sample B–XZ).

No	Sample ID	Diameter (mm)	Max. Load (kN)	Rp0.2 (MPa) *	Rm (MPa)	A35 (%)
1	B (1)–XZ	3.81	4.5	274	398	4
2	B (2)–XZ	3.81	4.6	275	405	4
3	B (3)–XZ	3.80	4.7	276	418	4
**AVERAGE VALUES**				**275**	**407**	**4**

* Indicative Value.

**Table A5:** Tensile test results (Sample C–YZ).

No	Sample ID	Diameter (mm)	Max. Load (kN)	Rp0.2 (MPa) *	Rm (MPa)	A35 (%)
1	C (1)–YZ	3.84	4.6	259	398	4
2	C (2)–YZ	3.83	4.4	267	382	4
3	C (3)–YZ	3.74	Rejected due to early breakage			
**AVERAGE VALUES**				**263**	**390**	**4**

* Indicative Value.

**Table A6:** Tensile test results (Sample U–XYZ).

No	Sample ID	Diameter (mm)	Max. Load (kN)	Rp0.2 (MPa) *	Rm (MPa)	A35 (%)
1	U (1)–XYZ	3.85	4.3	251	369	4
2	U (2)–XYZ	3.73	4.3	258	393	4
3	U (3)–XYZ	Not valid specimen (no proper thread manufacturing)				
**AVERAGE VALUES**				**255**	**381**	**4**

* Indicative Value.
